# Bacteriuria amongst Pregnant Women in the Buea Health District, Cameroon: Prevalence, Predictors, Antibiotic Susceptibility Patterns and Diagnosis

**DOI:** 10.1371/journal.pone.0071086

**Published:** 2013-08-16

**Authors:** Morike Ngoe Mokube, Julius Atashili, Gregory Edie Halle-Ekane, George M. Ikomey, Peter M. Ndumbe

**Affiliations:** Faculty of Health Sciences, University of Buea, Buea, South-West Region, Cameroon; University of Calgary, Canada

## Abstract

**Background:**

Bacteriuria is associated with significant maternal and foetal risks. However, its prevalence is not known in our community.

**Objectives:**

This study was carried out to determine the prevalence and predictors of bacteriuria in pregnant women of the Buea Health District (BHD) as well as the antibiotic sensitivity patterns of bacterial isolates. It also sought to determine the diagnostic performance of the nitrite and leucocyte esterase tests in detecting bacteriuria in these women.

**Methods:**

An observational analytic cross-sectional study was carried out amongst pregnant women attending selected antenatal care centres in Buea. We recruited 102 consenting pregnant women for the study. Demographic and clinical data were collected using structured questionnaires. Clean catch midstream urine was collected from each participant in sterile leak proof containers. Samples were examined biochemically, microscopically and by culture. Significant bacteriuria was defined as the presence of ≥10^8^ bacteria/L of cultured urine. Identification and susceptibility of isolates was performed using API 20E and ATB UR EU (08) (BioMerieux, Marcy l'Etoile, France).

**Results:**

Significant bacteriuria was found in the urine of 24 of the 102 women tested giving a bacteriuria prevalence of 23.5% in pregnant women of the BHD. Asymptomatic bacteriuria was detected in 8(7.8%) of the women. There was no statistically significant predictor of bacteriuria. *Escherichia coli* were the most isolated (33%) uropathogens and were 100% sensitive to cefixime, cefoxitin and cephalothin. The nitrite and leucocyte esterase tests for determining bacteriuria had sensitivities of 8%, 20.8% and specificities of 98.7% and 80.8% respectively.

**Conclusion:**

Bacteriuria is frequent in pregnant women in the BHD suggesting the need for routine screening by urine culture. Empiric treatment with cefixime should be instituted until results of urine culture and sensitivity are available. Nitrite and leucocyte esterase tests were not sensitive enough to replace urine culture as screening tests.

## Introduction

Bacteriuria is the presence of bacteria in the urine. Bacteriuria is said to be significant in the presence of ≥10^8^ colony forming units (CFU)/L. The danger with bacteriuria is that it does not always present with symptoms [1]. Occult infection occurs in about 2–7% of pregnancies and 30–40% of cases develop acute pyelonephritis later in pregnancy [2]. Also there are associations between maternal complications of pregnancy and pyelonephritis including hypertension, preeclampsia, anaemia, amnionitis, and endometritis [3]. Pyelonephritis can lead to renal scarring, hypertension and renal failure in the long run [4].

In pregnancy pyelonephritis increases the risk of preterm labour and delivery which result in prematurity and low birth weight with high perinatal morbidity and mortality. As an example, a review of over 50,000 pregnancies between 1959 and 1966 showed that women with bacteriuria and/or pyuria (no comment on the presence or absence of symptoms) in the last two weeks of pregnancy had a higher rate of perinatal mortality from a variety of causes when compared to non-infected women [2]. Another study showed that asymptomatic bacteriuria was independently associated with preterm delivery, hypertensive disorders, recurrent abortions, intrauterine growth restriction, polyhydramnios and oligohydramnios, premature rupture of membranes and labour induction [5]. There is also an increase in the risk of developing pyelonephritis in the puerperium [6].

The most common pathogen involved in bacteriuria is *Escherichia coli* accounting for 60 to 90% of infections in women. Other bacteria involved include *Klebsiella pneumoniae, Proteus mirabilis, and Pseudomonas aeruginosa*. These are all Gram-negative organisms and account for about 71.5% of all cases of bacteriuria [7]. Gram-positive organisms like *Staphylococcus saprophyticus* also cause bacteriuria [6].

Screening for asymptomatic bacteriuria has been included as one of the most cost-effective strategies for improving maternal and neonatal health in developing countries in a detailed analysis of interventions to achieve the Millennium Development Goals (MDGs) for health [8]. The adverse effects of undiagnosed bacteriuria have prompted the vast use of urine culture screening for all pregnant women attending antenatal clinics [9]. Other methods of diagnosing bacteriuria like dipstick nitrite and leucocyte esterase tests have a sensitivity of 82.9% and negative predictive value of 98.8% [10] when used together. However in Cameroon, Buea inclusive, culture is expensive and dipstick screening tests routinely used in pregnancy tend to focus on the presence of glucose and protein in urine rather than bacteriuria. As a result of this the prevalence and predictors of bacteriuria in pregnant women of the Buea Health District (BHD) are not known despite the noxious effects the former may have on the health of mothers and newborns.

In a study carried out in Bamenda, Cameroon, bacteriuria was found to be more prevalent in HIV-positive individuals (20.4%) compared with HIV-negative people (3%) [11]. However, this study was not specific to pregnant women. Also, predisposing factors other than HIV were not considered.

In this study we assessed the prevalence and predictors of bacteriuria in pregnant women in the BHD. We also sought to determine the sensitivity and specificity of the nitrite and leucocyte esterase dipstick tests which are more available, easy to implement and less expensive.

Furthermore, because worldwide resistance to antibiotics is increasing, fostered by indiscriminate use of empiric therapy in settings such as ours [12] in this study we also identify the causative organisms of bacteriuria in pregnant women in the BHD and their antibiotic susceptibility.

## Methods

An observational analytic cross-sectional study was carried out in July 2012 at selected health facilities (Buea Regional Hospital Annex, Buea Town, Buea Road, Muea and Mile 16 health centres) in Buea, Cameroon. The BHD is divided into seven health areas which have about 25 health facilities (public and private). Our study comprised 102 pregnant women of the BHD aged 15–45 years (reproductive age group) attending antenatal clinics at the selected health facilities and who had not been on antibiotics for at least 48 hours, were not in a state of emergency and consented voluntarily to participate. Selection was by convenient consecutive sampling.

### Data and Specimen collection

Cysteine lactose electrolyte deficient (CLED) agar (Laboratorios Conda, S.A.) was prepared as specified by manufacturers and dispensed unto 102 petri dishes before commencing participant recruitment. Prior to sample collection, socio-demographic and clinical data were collected by face-to-face administration of structured questionnaires.

Participants were advised to collect a clean catch of 10–20 ml of midstream urine using sterile disposable leak proof containers respecting aseptic collection techniques. Aliquots of urine samples were centrifuged at 500G for five minutes. Sediment from each sample was used to streak culture media and prepare a wet mount for microscopy. The remainder of the urine was tested biochemically using dipsticks impregnated with leucocyte esterase and nitrite tests. The urine dipstick kit used was Human-Combina 11S (Human Diagnostics mbH Max-Planck-Ring 21-D-65205, Wiesbaden, Germany). All samples were cultured so as to determine the sensitivities and specificities of the dipstick tests. Urine was cultured within two hours of collection to maximize recovery of pathogens. Culture plates and slides were observed by two laboratory technicians.

### Processing Samples

Growth was considered significant in the presence of ≥10^8^ CFU/L. Colonies of plates with significant growth were classified Gram-positive or negative. Genera and species of bacteria were identified using the Analytic Profile Index (API) 20E system (BioMérieux, 69280 Marcy-l'Etoile, France).

Antibiotic susceptibility testing was done using the ATB UR EU (08) strips (BioMérieux, 69280 Marcy-l'Etoile, France). Antibiotics were tested either at single or two concentrations. The antibiotics tested were: ampicillin (8 mg/l), ticarcillin (8 mg/l), piperacillin (8 mg/l), amoxicillin+clavulanic acid (8/2 mg/l) and piperacillin+tazobactam (8/4–16/4 mg/l), cephalothin (8 mg/l), cefixime (1 mg/l), cefoxitin (8 mg/l), ceftazidime (1–8 mg/l), cefotaxime (1 mg/l), cefuroxime (4 mg/l), cefepime (1–8 mg/l), imipenem (2 mg/l), fosfomycin (32 mg/l), gentamicin (2 mg/l), tobramycin (2 mg/l), amikacin (8 mg/l), nalidixic acid (24 mg/l), norfloxacin (0.5 mg/l), ofloxacin (0.5 mg/l), ciprofloxacin (0.5–1 mg/l), levofloxacin (1 mg/l) cotrimoxazole (2/38 mg/l) and nitrofurantoin (64 mg/l). For those tested at a single concentration, clarity signified sensitivity whereas turbidity indicated resistance. For those tested at double concentrations, clarity at both concentrations meant sensitivity, turbidity at both concentrations denoted resistance and clarity/turbidity pairs depicted intermediate susceptibility. All samples were analysed following the manufacturer's guidelines.

### Statistical Analysis

Data were keyed into the EPI info version 3.5.1 database (CDC/WHO, Atlanta, USA). Questionnaire data were systematically checked for errors during data entry by using legal values and specified ranges in Epi-info. In addition 10% of questionnaires were double-checked by a co-investigator, different from the original data entry person. Prior to analysis proper, the frequencies and ranges of every variable were verified for consistency with the study population. Analysis was done using EPI info version 3.5.1 and Microsoft Excel 2007 software.

To determine predictors of bacteriuria, odds ratios were calculated using the maximum likelihood estimation technique. Chi-square or Fisher's exact tests were used, as appropriate, to assess differences in the proportions of culture positive and negative participants. Exposure variables (age, level of education, parity, gravidity, marital status and the presence or absence of certain clinical features of urinary tract infection (UTI)) with p -values <0.25 were further tested via multiple logistic regression according to the method suggested by Bursac *et al* [13] to account for potential confounding. Associations with p-values <0.05 were considered statistically significant and exposure variables showing such association would have been considered predictors of bacteriuria in pregnancy. Adjusted odds ratios were also obtained by logistic regression. The sensitivities and specificities of the nitrite and leucocyte esterase test were calculated using standard methods as described by Kacmaz *et al* [14].

### Ethical considerations

Ethical approval to conduct the study was obtained from the Institutional Review Board (IRB) of the Faculty of Health Sciences, University of Buea, Cameroon. All participants provided written informed consent. With approval from the IRB, pregnant women aged less than the legal age of consent in Cameroon (21 years) and attending antenatal care services were considered emancipated minors and thus allowed to provide consent for themselves. Apart from the inconvenience of taking time to answer the research questionnaire, participants were not exposed to any undue risk. Individual results were provided to participants for their appropriate management.

## Results

The characteristics of the 102 pregnant women who participated in our study are summarized in Table 1. Most of our participants (68%) were aged 21–30 years, had attained secondary school (44.6%), were married (66%), multigravidas (54.9%) and in the third trimester of pregnancy (63.7%).

**Table 1:** Characteristics of 102 participants included in a study of bacteriuria in pregnancy in the Buea Health District, Cameroon.

Characteristic	Level	n	%
Age (years)	<21	15	14.7
	21–30	69	67.6
	31–40	17	16.7
	41–50	1	1
Marital status	Married	67	65.7
	Unmarried	35	34.3
Level of education	Primary	28	27.7
	Secondary	45	44.6
	University	29	27.7
Gravidity	Primigravida	31	30.4
	Multigravida	56	54.9
	Gravidity ≥5	15	14.7
Trimester	First	2	2
	Second	35	34.3
	Third	65	63.7

Of the 102 pregnant women tested, 24 had significant bacteriuria giving a prevalence of 23.5%.The overall prevalence of bacteriuria in women who were asymptomatic was 7.8%.

Table 2 shows the association between clinical characteristics and bacteriuria in pregnant women of BHD. Following multivariate analysis, we did not identify any statistically significant predictor of bacteriuria in pregnancy. However, women who had been pregnant at least two times (OR = 2.4; 95%CI = 0.3–17.5) and put to birth at least once (OR = 1.5; 95%CI = 0.3–8.1) tended to be more likely to have bacteriuria when compared to those who did not meet these criteria (Table 2).

**Table 2:** Association between clinical factors and bacteriuria in pregnancy in 102 participants in the Buea Health District, Cameroon.

Variable	Bivariate Analysis		Multivariate Analysis	
	Odds Ratio (95% CI)	P-values	Adjusted Odds Ratio* (95% CI)	P-values
Gravidity		0.02		0.4
Primigravida	1.0		1.0	
Multigravida	3.8(1.1–17.6)		2.4(0.3–17.5)	
Parity		0.06		0.6
Nullipara	1.0		1.0	
Primi/Multipara	3.1(1.1–10.0)		1.5(0.3–8.1)	
Fever		0.18		0.5
No	1.0		1.0	
Yes	0.46(0.08–2.5)		0.5(0.1–2.7)	

* : Adjusted for age, marital status and trimester.

**Figure 1:**
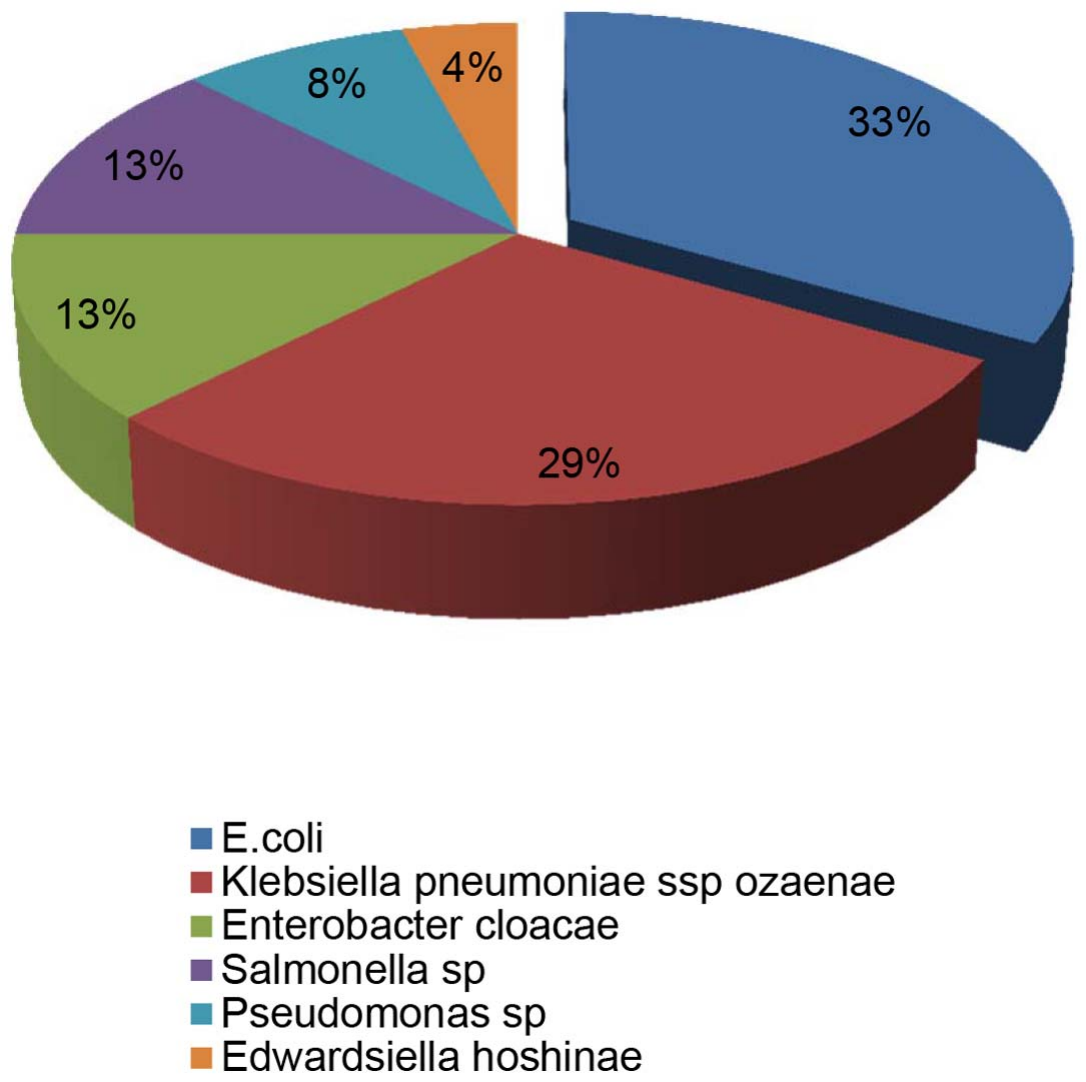
Uropathogens amongst 24 pregnant women with bacteriuria in Buea, Cameroon.

The most commonly isolated organism was *E. coli* (33%), followed by *Klebsiella pneumoniae sp* (29%). Other isolates were *Enterobacter cloacae* (13%), *Salmonella sp* (13%), *Pseudomonas sp (*8%), *Edwardsiella hoshinae* (4%), (Figure 1).

**Figure 2:**
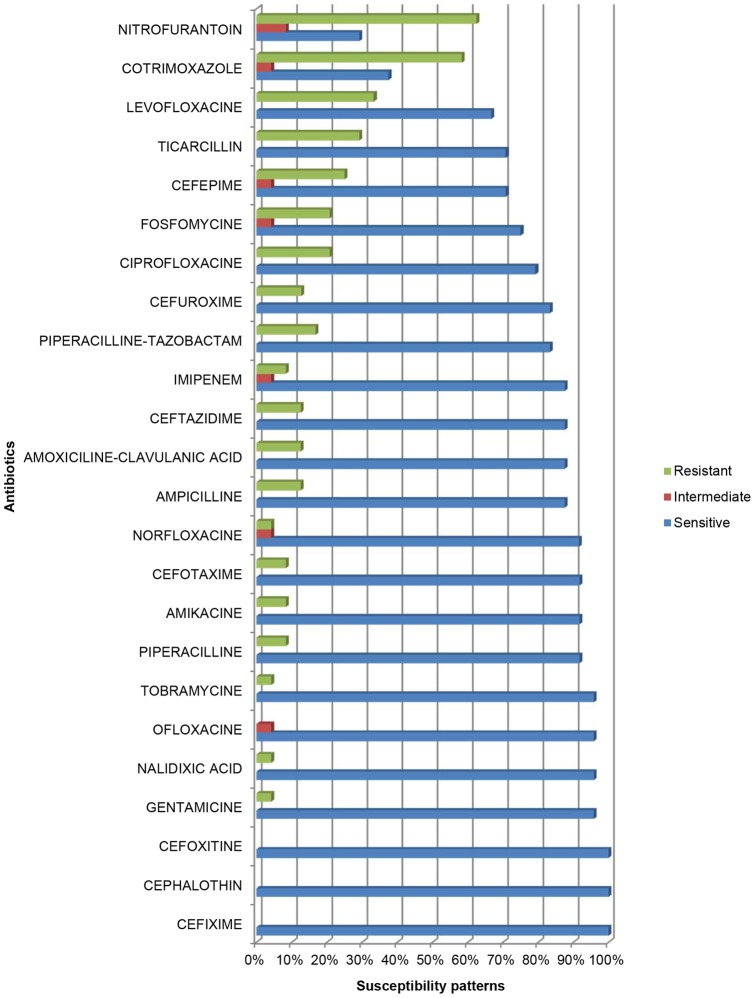
Antibiotic susceptibility patterns of bacterial isolates from 24 pregnant women with bacteriuria in Buea, Cameroon.

Overall, isolates were most sensitive (100%) to the cephalosporins like cefixime, cefoxitine, cephalothin. The next group were the aminoglycosides (gentamicin and tobramycin), then the fluoroquinolones nalidixic acid and ofloxacin each having sensitivities of 95.8%. Others with considerable sensitivity (91.7%) were piperacillin+tazobactam and ampicillin. The least sensitive were cotrimoxazole (37.5%) and nitrofurantoin (29.2%) (Figure 2).

The nitrite and leucocyte esterase tests had sensitivities of 8%, 20.8% and specificities of 98.7% and 80.8% respectively for detecting bacteriuria in pregnancy (Table 3).

**Table 3:** Sensitivity, specificity, predictive values of dipstick tests compared to urine culture in diagnosing bacteriuria in pregnancy in the Buea Health District, Cameroon.

Dipstick Test	Sensitivity	Specificity	PPV	NPV
Nitrite	8%	98.7%	67%	77.8%
Leucocyte esterase	20.8%	80.8%	25%	77%

PPV: Positive Predictive Value; NPV Negative Predictive Value.

## Discussion

In this study, we report a high prevalence of bacteriuria in pregnant women of the BHD. We did not identify any statistically significant predictor of bacteriuria. *Escherichia coli* were the most commonly isolated uropathogens. All pathogens were most sensitive to cefixime, cephalothin and cefoxitin. The sensitivities and specificities of the dipstick tests were very low.

The prevalence of bacteriuria in pregnant women of the BHD was 23.5%. This prevalence is higher than that reported by Assefa *et al* [15] and lower than that obtained by Okonko *et al* [16].

In addition, we had a 7.8% prevalence of asymptomatic bacteriuria in our study population. Our value falls in the range of 2–10% reported elsewhere [2], [17], [18], [19], [20], [21], [22]. Conversely, it is lower than that reported by other authors [1], [23], [24], [25], [26] Furthermore, the prevalence of asymptomatic bacteriuria (>2%) obtained from our study suggests that routine screening could be cost-effective in accordance with an American cost evaluation [27]. Variation in studies could be attributed to differences in gestational age, geographical location, socioeconomic status, setting of study (primary care, hospital, community) and variation in screening tests.

Predictors of bacteriuria in pregnancy identified in other studies [1], [18], [20], [21], [24], [28] are past history of UTI, age, anaemia, third trimester, level of education, low socioeconomic status, level of immunosuppression in people living with HIV/AIDS (PLWHA), gravidity. In our study, we did not identify any statistically significant predictor of bacteriuria in pregnancy. This agrees with findings made by Kehinde *et *al [25] and Andabati and Byamugisha [26]. In our study those with fever were less likely to have bacteriuria than those without fever. This could be because fever was due to more common causes such as malaria or due to infection with fastidious organisms unable to grow on our culture media.

*Escherichia coli* was the most common uropathogen isolated (33%). This correlates with findings made by Akoachere *et al* in Buea [29]. It is also consistent with findings in most studies [1], [16], [18], [21], [22]. On the other hand in a study conducted by Amadi *et al* [23], *Staphylococcus aureus* was the most prevalent uropathogen. In the same vein, a study carried out by Akoachere *et al* [29] in Bamenda found that *Klebsiella oxytoca* was the most prevalent organism. Variation in geographical location can account for these differences. *Klebsiella pneumoniae* was the second most prevalent pathogen in this study; a finding which is similar to that reported by recent studies in Sudan and Ethiopia [30], [31]. In this study there was a considerable prevalence (13%) of non-typhoidal *Salmonella sp*. This could be due to pregnancy and contamination. Naturally, the urethra of females is closer to the anus than in male [32]. Moreover, in pregnant women, the distension of the abdomen makes anal cleaning more cumbersome. Despite aseptic collection techniques expounded to participants, contamination is a possibility because participants were not supervised when collecting their urine. However, some of the participants with non-typhoidal *Salmonella* bacteriuria had symptoms suggestive of UTI (loin pain and fever) thus reducing the likelihood of contamination.

Most of the uropathogens isolated in our study, were most susceptible to cephalosporins such as cefixime, cefoxitin and cephalothin. Isolates identified by Njunda et al [9] also showed a high sensitivity to another cephalosporin: ceftriaxone. This is unlike other studies which state that isolates had a better sensitivity to fluoroquinolones such as ciprofloxacin [1], [24], and fosfomycin [22]. It is worth noting that the administration of cephalosporins is relatively safer during pregnancy as compared to fluoroquinolones which are contraindicated unless there are no other alternatives [33].

On the other hand nitrofurantoin and cotrimoxazole had the lowest sensitivities in our study. This correlates with findings by Akoachere et al [29] and differs from those by Okonko et al [34], whose isolates had a good sensitivity to nitrofurantion. The resistance to cotrimoxazole in our study can be explained by its widespread over-the-counter use in our locality. Apparently, isolates exhibited considerable susceptibility to antibiotics used for empirical treatment of UTI in pregnancy in the BHD like ampicillin and gentamicin. The considerable susceptibility to gentamicin correlates with findings made by Akoachere et al in Buea [29].

There is a variation in the sensitivity and specificity of dipstick tests. Jayalakshmi *et* *al* [10] got a leucocyte esterase test sensitivity of 61.7% and a specificity of 92.7%. The nitrite test had a higher sensitivity and specificity. When both tests were used in synergy, the sensitivity was 53.1% and the specificity very high (100%). Kacmaz *et* *al* [14], had sensitivities and specificities of 60% and 99.2%, 70% and 92.5% of nitrite and leucocyte esterase tests respectively.

Our study yielded a very low sensitivity of both nitrite and leucocyte esterase tests (8% and 20.8% respectively). The lower sensitivity of nitrite tests compared to previous reports [10], [14], [35] may be due to the fact that this test is more accurate when the first voided specimen is collected or when urine has been stored in bladder for over four hours. It was cumbersome to ensure such conditions in our study. False negatives can also occur when the organism present does not produce nitrate reductase and when dietary nitrate is absent [14]. The low sensitivity of the leucocyte esterase test could be due to the fact that about 15% of those who tested positive did not have bacteriuria. This could be associated with fastidious organisms that cause sterile pyuria like *Chlamydia trachomatis*, *Neisseria gonorrheae* [10].

In our study, identification of predictors of bacteriuria in pregnancy is limited by the relatively small sample size. Also, not all the factors which could possibly affect bacteriuria in pregnancy were assessed. Factors like anaemia, socioeconomic status, level of immune depression in people living with HIV/AIDS were not assessed. Furthermore, pregnant women with asymptomatic bacteriuria were not followed up to determine their risk of developing overt bacteriuria and other adverse outcomes.

Besides, our study is limited to just one health district in Cameroon. Nevertheless, our findings are consistent with other studies [9], [19], [20], [21], [22], [26], [29]. The decentralized health system makes it important to provide district-level specific evidence-based recommendations.

Therefore we suggest that further research be conducted incorporating factors which we did not evaluate in order to determine predictors of the condition in our locality and in other health districts of Cameroon. Predictors could be incorporated into screening and testing algorithms in order to decrease overall costs while improving maternal and infant outcomes. Further research should also be carried out to determine the risk of asymptomatic bacteriuria progressing to UTI which causes other unwanted outcomes such as preterm deliveries and low birth weight.

In conclusion, this study reveals that about one out of every four pregnant women in the BHD may have bacteriuria. This is worrying as bacteriuria in pregnancy could be very harmful. There was no statistically significant predictor of bacteriuria thus limiting the possibility of targeted screening. The most common uropathogen isolated from pregnant women was *Escherichia coli*. Isolates were most susceptible to cefixime, cefoxitin, cephalothin and showed considerable sensitivity toward empirically used antibiotics like ampicillin and gentamicin. Additionally, dipstick tests did not prove to be sensitive or specific enough to replace culture as a routine test.
